# Patency of Drug-Coated versus Conventional Balloon Angioplasty for Hemodialysis Access Stenosis

**DOI:** 10.5334/jbsr.3315

**Published:** 2023-12-21

**Authors:** Thanawat Anukanchanavera, Keerati Hongsakul, Phurich Janjindamai, Surasit Akkakrisee, Kittipitch Bannangkoon, Sorracha Rookkapan, Ussanee Boonsrirat, Sarayut Geater

**Affiliations:** 1 Prince of Songkla University, Thailand

**Keywords:** drug-coated balloon angioplasty, conventional balloon angioplasty, primary patency, dialysis access stenosis, end-stage renal disease

## Abstract

**Purpose:**

This study aimed to evaluate the post-intervention target primary patency of drug-coated balloon angioplasty (DCBA) compared with conventional balloon angioplasty (CBA) in the treatment of the dysfunctional autogenous arteriovenous fistula (AVF) in a real-world clinical setting.

**Materials and Methods:**

This retrospective study included 24 patients with end-stage renal disease, who developed dysfunctional AVF during hemodialysis, and underwent endovascular treatment using CBA and DCBA from January 1, 2014, to June 30, 2021. The demographic data of patients and details regarding their fistula were recorded. Post-intervention target primary patency was analyzed.

**Results:**

Sixteen men and 8 women with an average age of 63.9 ± 14.2 years, who underwent 333 endovascular treatments in 57 target lesions of access were enrolled. DCBA was a protective factor for the treatment of a target lesion of dysfunctional access with an adjusted hazard ratio of 0.725 (95% confidence interval [CI]: 0.528–0.996; P = 0.047). According to the Weibull proportional hazards regression model, DCBA showed a longer post-intervention target primary patency than CBA.

**Conclusion:**

DCBA has better outcomes in terms of post-intervention target primary patency in the real-world treatment of dysfunctional autogenous AVF.

## Introduction

The incidence of end-stage renal disease (ESRD) has been increasing recently, and the advent of vascular access site as an autogenous arteriovenous fistula (AVF) is a renal replacement therapy that improves survival in patients with this disease [1].

Percutaneous transluminal angioplasty is a standard primary treatment for AVF dysfunction for improving its durability [2,3]. According to the 2019 clinical practice guideline for vascular access, conventional balloon angioplasty (CBA) is a reasonable primary treatment for the stenotic segment of vascular access. On the other hand, drug-coated balloon angioplasty (DCBA) is another treatment option for recurrent stenotic lesions and reportedly has better patency rate outcomes than CBA [4].

Patients undergoing hemodialysis with AVF usually have multiple episodes of access stenosis, which are treated with multiple CBAs before a DCBA. Despite performing DCBA for early significant restenotic lesions, recurrence of these lesions necessitates repeated treatment with CBAs or DCBAs in real-world clinical practice; these are also performed in the same patients with the same lesions. Many previous publications have reported that compared with CBA, DCBA demonstrated better outcomes in terms of patency rate than CBA; however, only a few studies have regarded intra-individual control and analyzed data in actual real-world data [5,6].

Therefore, this study aimed to compare the post-intervention primary patency rate between DCBA and CBA for treating dysfunctional AVF in a real-world clinical setting.

## Materials and Methods

### Patients

The Ethics Committee of our institution approved this retrospective single-center study (IRB 64-295-7-4). Data were collected from the database and picture archiving and communication system of our hospital, which is a university hospital in southern Thailand, between January 1, 2014, and June 30, 2021. We included 27 patients with mature AVFs undergoing hemodialysis with AVF dysfunction, who had a history of receiving endovascular treatment by CBA and DCBA. The indication for treatment in these patients was dysfunctional AVFs with significant stenosis (≥ 50% luminal stenosis of access) from the initial angiogram. DCBA was supplementary used in cases of early significant restenosis of access. However, three patients were excluded due to incomplete data. Additionally, any stenotic lesions that were treated using a special device, such as a cutting balloon or stent placement, were also excluded. Ultimately, 24 consecutive patients were enrolled.

### Procedure

Informed consent was obtained from all patients before the procedure. A standard protocol of the interventional radiology unit was used for the endovascular procedures, which were performed under local anesthesia. Doppler color ultrasound was initially conducted from the arteriovenous anastomosis to the cephalic arch to evaluate the site and degree of stenosis and to identify a cannulation site for vascular sheath insertion. In cases of significant access stenosis, a 6 or 7 French (Fr) vascular sheath was inserted antegradely or retrogradely, depending on the site of stenosis. The initial angiogram via the vascular sheath was done to identify the stenotic site of the arteriovenous access and central vein. Subsequently, 1,500 units of heparin was administered, followed by the insertion of a 0.035-inch hydrophilic guidewire with a high-pressure, non-compliant balloon catheter (Conquest, BARD Medical, AZ, USA or Mustang, Boston Scientific, MA, USA) with a diameter of 6–9 mm via the vascular sheath across the stenotic site. CBA was performed at the stenotic site with a nominal or more than nominal pressure of full balloon expansion for two minutes. A final angiogram was performed to check for residual stenosis or complications. In cases with a history of early significant restenosis of target lesion, a CBA was first performed, which was followed by a DCBA if the residual stenosis of the target lesion was <30%. Drug-coated balloon catheters (IN.PACT™ Admiral™, Medtronic, MN, USA), which are 1 mm larger in diameter than conventional balloon catheters (7–10 mm), were advanced via a 0.035-inch hydrophilic guidewire and placed at the stenotic site. It was inflated up to a normal pressure of 8 atmospheres and retained for three minutes. A post drug-coated balloon angiogram was performed to evaluate its patency and any complications.

For the central vein lesion, an 8 or 9 Fr vascular sheath was inserted into the basilic or cephalic vein of the arm. A 0.035-inch hydrophilic guidewire with high pressure non-compliant balloon catheter (Conquest or Atlas; BARD Medical, AZ, USA) with a diameter of 12 or 14 mm was advanced via the vascular sheath. A CBA was performed in significant stenotic target lesions with a two-minute retention of the fully expanded balloon. DCBAs in the central veins were performed after a CBA in cases of early significant restenosis, using 12- (IN.PACT™ Admiral™, Medtronic, MN, USA) or 14-mm (Elutax, Aachen Resonance, Aachen, Germany) balloon catheters. A final angiogram was performed to evaluate the patency and complications.

Upon completing the procedure, the vascular sheath was removed, and manual compression was performed until hemostasis was achieved.

### Follow-Up

All patients who had a successful endovascular procedure underwent regular hemodialysis and attended follow-up at the hemodialysis center and vascular surgery clinic. Patients who had AVF dysfunction detected during hemodialysis and/or clinical evaluation were examined and referred to the interventional radiology unit for endovascular treatment.

### Definitions

According to the standardized definitions for hemodialysis vascular access [7,8], early significant restenosis was defined as a stenosis of ≥50% of the lumen within three months after previous CBA. Procedural success was defined as a post-dilatation angiogram showing <30% residual luminal diameter stenosis with a palpable thrill. Post-intervention target primary patency was defined as the interval between the procedure and the first subsequent intervention on the target lesion or for access thrombosis. Major complications were defined as those requiring additional treatment, those with permanent sequelae, or death. Minor complications were defined as problems requiring no or minimal therapy and with no sequelae.

### Statistical analysis

For continuous and categorical data, baseline characteristics are reported as mean ± standard deviation and number (%), respectively. Repeated time-to-event risk set is defined as the total amount of time from the beginning of analysis until catheter removal [9]. Hazard ratio (HR) was calculated using mestreg or multilevel mixed-effect parametric survival regression [10]. The Weibull hazard distribution was selected after analyzing alternative distribution models with the lowest Akaike and Bayesian information criteria values. Model-derived survival curves were created to illustrate the survival odds of each treatment type. Statistical significance was set at a *P* value of < 0.05.

## Results

This study included 24 patients with ESRD with an average age of 63.9 ± 14.2 years and who are most commonly men (16 cases). Hypertension was the most common underlying disease (22 cases). Difficulty in dialysis, poorly palpable thrill, and venous hypertension were observed in eight cases each. The most common type of AVF dysfunction was radiocephalic AVF (15 cases). Endovascular interventions were performed 333 times for 57 stenotic lesions. The juxta-anastomotic lesion was the most common site and received the highest number of treatments (48.95%); 78.08% of treated lesions had a severe degree of stenosis, and CBA was the most common procedure used in this study (85.59%). The demographic and procedural data are summarized in Table 1.

**Table 1:** Demographic and procedural data of 24 patients with 57 lesions.


PARAMETER	NUMBER (%)

**Age**	63.9 ± 14.2 years

**Sex**	

Men	16 (66.67)

Women	8 (33.33)

**Underlying disease**	

Hypertension	22 (91.67)

Dyslipidemia	15 (62.50)

Diabetes mellitus	10 (41.67)

**Presenting symptom**	

Dialysis difficulty	8 (33.33)

Poorly palpable thrill	8 (33.33)

Venous hypertension	8 (33.33)

**Type of arteriovenous dysfunction**	

Radiocephahic type	15 (62.50)

Brachiocephalic type	9 (37.50)

**Number of treatments per location of treated lesion**	

Juxta-anastomosis	163 (48.95)

Central veins	91 (27.33)

Venous outflow	54 (16.22)

Cephalic arch	21 (6.30)

Arteriovenous anastomosis	4 (1.20)

**Degree of stenosis of target lesions**	

Moderate stenosis	66 (19.82)

Severe stenosis	260 (78.08)

Complete occlusion	7 (2.10)

**Number of procedures performed**	

Conventional balloon angioplasty	285 (85.59)

Drug-coated balloon angioplasty	48 (14.41)


Procedural success was found in 333 procedures (100%). Minor complications occurred in eight cases, including two cases of vascular spasm, two minimal contrast leakages, and four focal dissections. All minor complications were successfully treated using balloon tamponade, and no major complications occurred.

**Figure 1:**
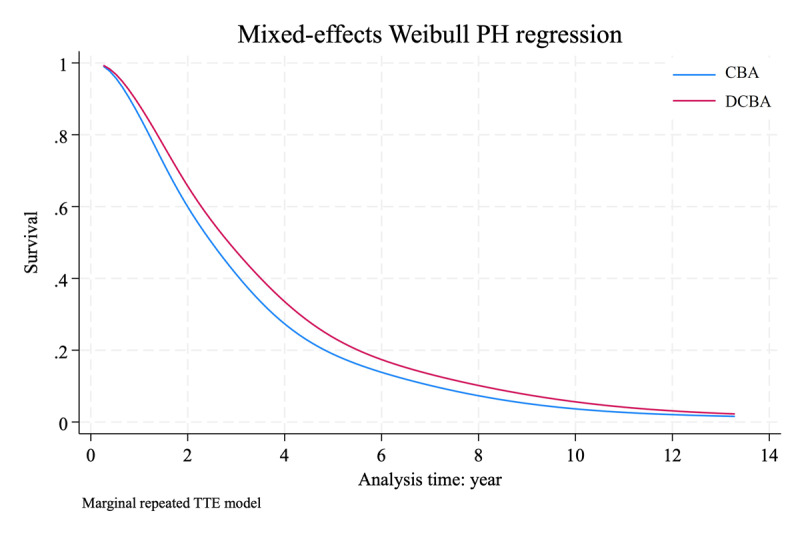
Comparison of the post-intervention target primary patency between CBA and DCBA using Weibull PH regression. PH, proportional hazards; CBA, conventional balloon angioplasty; DCBA, drug-coated balloon angioplasty; TTE, time-to-event.

The multilevel mixed-effect parametric survival regression is presented in Table 2. The HR of the DCBA was 0.735 (95% confidence interval [CI]: 0.536–1.010; P = 0.058). After considering the degree of target lesion stenosis as an adjusted predictive factor, the adjusted HR was 0.725 (95% CI: 0.528–0.996; P = 0.047). According to the Weibull proportional hazards regression, the post-intervention target primary patency of the DCBA and CBA at 6 months, 1, 2, and 3 years were 97%, 88%, 64%, and 45% as well as 95%, 84%, 58%, and 39%, respectively (Figure 1).

**Table 2:** Comparison between conventional and drug-coated balloon angioplasties using the Weibull hazard distribution.


TECHNIQUE	HAZARD RATIO (95% CI)	*p*-VALUE	ADJUSTED HAZARD RATIO (95% CI)	*p*-VALUE

**CBA**	1		1	

**DCBA**	0.735 (0.536–1.010)	0.058	0.725 (0.528–0.996)	0.047

**Degree of lesion stenosis**				

Moderate (50%–69%)			1.499 (0.493–4.557)	0.476

Severe (70%–99%)			1.859 (0.630–5.484)	0.261

Complete occlusion (100%)			1.996 (0.477–8.349)	0.344


CI, confidence interval; CBA, conventional balloon angioplasty; DCBA, drug-coated balloon angioplasty.

## Discussion

In this study, the post-intervention primary patency of the target lesions treated with DCBA was better than that in those treated with CBA in the real-world clinical management of dysfunctional AVFs. Our results are similar to those of previous studies.

Endovascular treatment with DCBA is accepted and widely used for treating coronary and peripheral arterial diseases [11,12]. Similarly, previous studies have demonstrated better patency after DCBA compared with CBA for the treatment of dialysis access stenosis [13,14]. Two randomized controlled trials (RCTs), conducted by Katsanos et al. [15] and Lookstein et al. [16], showed the significantly superior six-month primary target patency of DCBA compared with CBA as 70% versus 25% and 82.2% versus 59.5%, respectively. Additionally, an RCT by Irani et al. [14] reported that DCBA had better primary lesion patency at six months and one year compared with CBA (81% vs. 61% and 51% vs. 34%, respectively). Moreover, a systematic review and meta-analysis by Yanwee et al. [13] showed that DCBA had better outcomes in terms of the six- and twelve-month primary patency compared with CBA when used to treat dialysis access stenosis.

Similar to previous reports, this study also showed that DCBA provided superior patency of the target lesion in the AVF compared with CBA [13,14,15,16,17]. According to the 2019 clinical practice guideline for vascular access [4], DCBA was not recommended as a primary treatment option for hemodialysis access stenosis but was recommended for early significant restenosis of access. However, in real-world practice, the use of DCBA and CBA varies depending on the situation and opinion of the individual operator. For the same patient with the same lesion, CBA may be performed first as standard practice; however, if this patient develops early significant restenosis of hemodialysis access, either CBA or DCBA could be used for treatment. Moreover, a restenosis of access after DCBA can be re-treated with CBA. Patients undergoing hemodialysis with AVF usually have multiple episodes of access restenosis, which are treated with multiple rounds of CBA and DCBA. Therefore, we used a model analysis of recurrent multiple events of significant AVF stenosis, which were treated with CBA and DCBA to create a scenario mimicking real-world practice. This statistical model stratified individual bias by distributing multiple time-to-events in the same patients with the same target lesions, which was beneficial for adjusting the confounding factors for each lesion [9,10]. According to our multilevel mixed-effect parametric survival regression, DCBA was a protective procedure compared with CBA. Additionally, our Weibull proportional hazards regression analysis revealed that DCBA had superior post-intervention primary patency compared with CBA. This may be due to the antiproliferative effect of paclitaxel on the venous wall.

A strength of this study was that this was the first study that analyzed post-intervention primary target patency using a model of recurrent multiple events, which was as close to real-world practice as possible. However, this study also had some limitations. First, this was a single-center retrospective study and included only a small number of participants; however, we corrected this issue by using the number of events for model analysis. Second, this study did not conduct a cost-effectiveness analysis between DCBA and CBA. Lastly, the efficacy of DCBA in relation to the location of stenosis was not conducted. These limitations should be investigated in future studies.

In conclusion, based on our model of recurrent multiple events, DCBA is superior to CBA in terms of prolonged post-intervention primary patency of the stenotic autogenous AVF in real-world practice.

## Data Accessibility Statement

The original data is available upon reasonable request to the corresponding author.
